# Assessing the impact of building footprint dataset choice for health programme planning: a case study of indoor residual spraying (IRS) in Zambia

**DOI:** 10.1186/s12942-025-00398-7

**Published:** 2025-05-24

**Authors:** Heather R. Chamberlain, Derek Pollard, Anna Winters, Silvia Renn, Olena Borkovska, Chisenga Abel Musuka, Garikai Membele, Attila N. Lazar, Andrew J. Tatem

**Affiliations:** 1 WorldPop, School of Geography and Environmental Science, University of Southampton, University Road, Southampton, SO17 1BJ UK; 2 Akros Research, 45 A Roan Road, Kabulonga, Lusaka, Zambia; 3 Akros Inc., 4302 Timberlane, Missoula, MT 59802 USA; 4 School of Public and Community Health Sciences, University of Montana, Missoula, USA; 5 GRID3 Inc., 211 East 43Rd Street, 7 Th Floor #219, New York, NY 10017 USA; 6 Blue Byte Analytics Ltd., Plot No. 609/E/48/B/4/2, Off Hybrid Road, Chamba Valley, Lusaka, Zambia; 7 Department of Geography and Environmental Studies, University of Zambia, Great East Road Campus, Lusaka, Zambia

**Keywords:** Building footprints, Malaria, Microplanning, Geospatial, Satellite imagery

## Abstract

**Background:**

The increasing availability globally of building footprint datasets has brought new opportunities to support a geographic approach to health programme planning. This is particularly acute in settings with high disease burdens but limited geospatial data available to support targeted planning. The comparability of building footprint datasets has recently started to be explored, but the impact of utilising a particular dataset in analyses to support decision making for health programme planning has not been studied. In this study, we quantify the impact of utilising four different building footprint datasets in analyses to support health programme planning, with an example of malaria vector control initiatives in Zambia.

**Methods:**

Using the example of planning indoor residual spraying (IRS) campaigns in Zambia, we identify priority locations for deployment of this intervention based on criteria related to the area, proximity and counts of building footprints per settlement. We apply the same criteria to four different building footprint datasets and quantify the count and geographic variability in the priority settlements that are identified.

**Results:**

We show that nationally the count of potential priority settlements for IRS varies by over 230% with different building footprint datasets, considering a minimum threshold of 25 sprayable buildings per settlement. Differences are most pronounced for rural settlements, indicating that the choice of dataset may bias the selection to include or exclude settlements, and consequently population groups, in some areas.

**Conclusions:**

The results of this study show that the choice of building footprint dataset can have a considerable impact on the potential settlements identified for IRS, in terms of (i) their location and count, and (ii) the count of building footprints within priority settlements. The choice of dataset potentially has substantial implications for campaign planning, implementation and coverage assessment. Given the magnitude of the differences observed, further work should more broadly assess the sensitivity of health programme planning metrics to different building footprint datasets, and across a range of geographic contexts and health campaign types.

**Supplementary Information:**

The online version contains supplementary material available at 10.1186/s12942-025-00398-7.

## Background

Health programme planning is an inherently geographic problem, requiring an understanding of populations and infrastructure to estimate where, when and what resources are required. From provision of specific services to planning national vaccination programmes, information on target populations and where they are located is key [1–3]. The COVID-19 pandemic brought health programme planning into the spotlight, with efforts needed to rapidly plan and implement programmes for patient care, testing and subsequently vaccination [4, 5]. Effective health programme planning for a range of communicable diseases, informed by an understanding of populations and infrastructure, far predates the COVID-19 pandemic though. Early documented examples include the 1854 London cholera outbreak, where the Broad Street pump was identified as a source of infection following collection of data on cases in relation to the resident population in the vicinity [6]. More recently, comprehensive mapping of settlements and tracking of vaccination teams to ensure high coverage rates of target populations, contributed to the elimination of wild polio virus in northern Nigeria [7–9].

To support a geographic approach to health programme planning, detailed data are needed to understand the spatial distribution of populations, infrastructure and the built and natural environments [1, 10]. In the early stages of COVID-19 mass vaccination campaigns, such data enabled assessment of population access to vaccination facilities and identification of where long travel times may be an impediment to a population being vaccinated [11–14]. Similar analyses have highlighted gaps in provision of health services [15–17] and vaccination programmes for many communicable diseases [18–20]. The availability, completeness and quality of high-resolution geospatial data on populations, settlements and infrastructure however varies between countries. In settings with well-developed geospatial data systems and comprehensive national address databases, authoritative data on the locations of residential populations may be readily available, whilst in other settings such data may be very limited or outdated, if it exists at all. Low-income countries tend to have less well-developed geospatial data systems and high burdens of communicable diseases, resulting in a high demand for planning health programmes and campaigns, but limited geospatial data to support this planning [21].

Developments in computing power, satellite imagery and machine-learning algorithms over the past decade have enabled the growth of new sources of geospatial data relevant for health programme planning, including increasingly detailed land cover [22–24], settlement [25–30] datasets at global and continental scales. In the past five years, advances in feature extraction algorithms, combined with availability of high-resolution satellite imagery, have enabled detailed mapping to the level of individual buildings at scale [31]. Several national and multi-country building footprint datasets have been published, predominantly by commercial companies such as Google (https://sites.research.google/open-buildings, Microsoft (https://www.microsoft.com/en-us/maps/building-footprints) and Ecopia (https://www.ecopiatech.com/global-feature-extraction). For many countries, these new datasets have provided building footprint data for the first time without extensive manual digitisation efforts, although licensing does vary between datasets, with only some openly published (e.g. Microsoft and Google building footprints). Prior to these developments, no national building footprint datasets from authoritative sources were openly available for many countries with high burdens of communicable diseases, including all countries in Africa [32].

The potential of new sources of high-resolution geospatial data in planning and delivery of health campaigns is increasingly being harnessed, supported by published guidance such as the Geo-Enabled Microplanning Handbook [33]. Geospatial datasets are also being integrated into user-friendly tools such as the Reveal platform (https://revealprecision.com), which supports health campaign planning and delivery by integrating geospatial data on building footprints, points of interest, and health and administrative boundaries into an interactive map interface [34–36]. This is accompanied by an interface to plan out campaigns including calculating commodity requirements and developing microplans (detailed plans for delivery of health interventions to target populations [37, 38]. The Reveal platform was initially developed to support malaria control programmes but has been used for a range of health interventions, including geo-enabled microplanning for vaccination campaigns and mass drug administration [39, 40].

Vector control measures are a mainstay of malaria control programmes, with both long-lasting insecticide-treated nets (LLINs) and indoor residual spraying (IRS) being widely used [41] to reduce vector abundance and minimise transmission. LLINs are intended to provide protection for household-members sleeping under a net, by physically preventing individuals from being bitten by infected mosquitoes whilst asleep. Insecticide-treated nets kill mosquitoes which land on the net, thus helping to reduce the vector population and providing some degree of community-level protection, in addition to the immediate household-level protection. In contrast, IRS does not prevent individuals from being bitten by an infected mosquito but instead is intended to kill endophilic mosquitoes that rest on walls of residential buildings after feeding, and in this way reduce the vector population and disrupt the malaria transmission cycle. For IRS to be effective in reducing malaria incidence in a community, high- and uniform-coverage is needed,a minimum threshold of 80% coverage of eligible structures is commonly used as an operational target [41].

For IRS and LLIN campaigns to reach the high levels of coverage needed, data on the count and location of households, residential buildings and population are required. The availability of such data can be limited and quickly become outdated. Surveys or household listing exercises to collect new data are expensive, time-consuming, and resource-intensive, but until recently were the sole option for updating such data. Manual identification and digitising of buildings from satellite imagery has been used as an alternative in the context of planning and monitoring IRS campaigns in Zambia, and was found to be 22 times faster and 10 times less costly than field-based enumeration [35]. High-resolution geospatial data on population, settlements and infrastructure, have been used extensively in studies on malaria risk, control and elimination [42–45], and are increasingly being used to support programme planning for malaria [34, 46–48]. The need to utilise good quality data to maximise malaria control impact through sub-national targeting, is emphasised in the WHO High Burden to High Impact (HBHI) initiative [48]. New building footprint data products can help in maximising coverage of IRS campaigns by estimating the volume of insecticide and number of spray teams required, supporting spray teams in locating buildings, enabling identification of missed buildings and subsequent calculation of coverage rates. They can also help in identifying remote communities that are hard to reach, and therefore often missed during health service delivery.^49^

The growth in building footprint data products has brought new opportunities for detailed geospatial data to be utilised in supporting health programmes, but the degree to which these building footprint data products are interchangeable has only recently started to be explored [31, 50]. Knowledge of the impact of utilising different building footprint data products in subsequent analyses and decision making remains limited. In this study, we explore the impact of utilising different building footprint data products in analyses to support health intervention planning. We focus on Zambia, with a case study on planning implementation of malaria vector control measures and in particular the identification of priority settlements for IRS campaigns. Our results show that when the same criteria are applied with different building footprint datasets, there is considerable variation in the number and location of priority settlements that are identified.

## Methods

To quantify the impact of utilising different building footprint datasets in health programme planning, we use the example of IRS campaign planning in Zambia. To identify priority locations for IRS campaigns, we employ target criteria used in recent campaigns, conducted by implementing partners of the National Malaria Elimination Programme (NMEP). The target criteria relate to the area of, distance between and count of building footprints, and we apply the same criteria with four different building footprint data products. We separately apply two methods to identify suitable settlements or clusters of building footprints that meet the target criteria for potential inclusion in IRS campaigns. The first approach utilises existing mapped data on settlement extents and the second derives clusters of structures, solely based on the building footprint data with a proximity threshold applied.

### Study setting

There were 7,050,968 malaria cases in Zambia in 2021, with a case incidence of 340/1000 population [51]. Malaria risk in Zambia is spatially heterogeneous, with high incidence particularly in the north, north-west and east of the country, as well as in some areas near national borders. The NMEP has used both IRS and LLINs as vector-control strategies in recent years. IRS was prioritised as a primary vector control approach alongside LLINs in the National Malaria Elimination Strategic Plan (NMESP) for 2017–2021. The current NMESP for 2022–2026 prioritises LLINs as the primary intervention for vector control, with IRS targeted in high burden areas [71].

Geospatial data on buildings has been used to plan vector control activities under both strategic plans. District IRS planning and implementation maps, which included counts of manually digitised building footprints and malaria risk data per settlement, were first used in 2014 in 15 districts in Luapula and Central provinces to prioritise available IRS resources [35]. In 2015 and 2019, these building footprint enumeration maps were expanded to 42 districts, across five provinces, to support IRS planning. Sources of building footprints used during this time included manual delineation and enumeration of buildings from satellite imagery, field-verified footprint data and OpenStreetMap. The development by Ecopia of the first multi-country building footprint dataset for sub-Saharan Africa in 2019, provided building footprints nationally for Zambia (under a licence permitting use for humanitarian purposes), without requiring bespoke exercises to manually digitise buildings. The Ecopia building footprints enabled counts of buildings and derived settlement extents [52] to be integrated into malaria microplanning maps for the first time in the 2020–21 vector control campaign. Subsequently, the development of open datasets of building footprints, has widened the availability of data further, with Google Open Buildings data then used in two districts to support IRS plannings in 2022 and 2023.

One of the challenges with using satellite-derived building footprint datasets is that footprints are not labelled as residential or non-residential structures. Without attribute data on building function or use, vector control interventions in Zambia have previously applied minimum and maximum area thresholds to exclude probable non-residential buildings [35]. Alternatively, the proportion of structures that were residential has been estimated, based on samples of field-verified data. These approaches have enabled estimation of counts of residential buildings per settlement and identification of priority settlements for IRS [34–36, 47].

### Data

In their most basic form, building footprints consist of mapped digital outlines of buildings (polygons) and provide information on the location, size and shape of buildings. Some building footprint datasets may also include additional attributes, such as a building’s type or height. They enable building and settlement locations to be mapped, and their proximity and spatial arrangement with neighbouring buildings understood [53]. Four different building footprint data products were included in the comparative analysis (Table 1): Google Open Buildings [54], Microsoft “Global” building footprints [55], Ecopia DigitizeAfrica building footprints [56] and buildings extracted from OpenStreetMap (OSM).

**Table 1:** Details of the building footprint data products available as of October 2023, and included in the comparative analysis for Zambia

	Dataset	Version; release date	Method; source	Notes
Ecopia	Digitize Africa building footprints	“Year 1”; published 2019	Automated feature extraction from satellite imagery from multiple years (Maxar 30-50 cm imagery) https://www.ecopiatech.com/	Custom, commercial license which restricts usage predominantly to humanitarian applications
	Digitize Africa building footprints	“Year 2”; published 2021		
Google	Open buildings	v2; published 2022	Automated feature extraction from satellite imagery from multiple years (Google Maps imagery with 50 cm spatial resolution) https://sites.research.google/open-buildings/	CC BY 4.0 License or ODbL (Open Database License) v1.0 Includes data at building level on precision confidence score
	Open buildings	v3; published 2023		
Microsoft	“Global” building footprints	2022–11–15; Downloaded January 2023	Automated feature extraction from satellite imagery from multiple years (Bing Maps imagery, including Maxar and Airbus) https://github.com/microsoft/GlobalMLBuildingFootprints	ODbL v1.0 License No clear versioning system
	“Global” building footprints	2023–10–16; Downloaded October 2023		
OpenStreetMap (OSM)	OSM features tagged building = *	Downloaded January 2023	Predominantly manual digitization from satellite imagery, aided by some automated feature detection (imagery from various sources) https://www.openstreetmap.org/	ODbL v1.0 License No versioning system as constantly being updated given VGI-nature of OSM
	OSM features tagged building = *	Downloaded October 2023		

Building footprints from Ecopia, Google and Microsoft are all examples of data products created by commercial companies, using automated feature extraction methods and high-resolution satellite imagery [57]. In contrast, OSM buildings are an example of volunteered geographic information (VGI) product, with features digitised by a community of mappers and added to OpenStreetMap. Over the past decade, billions of features globally have been added to OSM, but given its VGI-nature, inevitably is spatially heterogenous in terms of geographic coverage. Prior to the recent developments of multi-country building footprint datasets through automated feature extraction, OSM was the only source of mapped building data available for many countries. A third potential source of building footprints is authoritative datasets from government agencies,however no authoritative national dataset of building footprints is available for Zambia [32].

Given the rapidly evolving data landscape, two versions of each building footprint data product were considered (eight datasets in total: Table 1). All building footprint data products consisted of vector polygons with limited, if any, additional attribute information. Of the three building footprint data products generated through feature extraction from satellite imagery, only Ecopia includes the reference date of the satellite imagery as a feature attribute. Imagery dates are not specified for the Google or Microsoft building footprints, but most likely span multiple years. Some information related to building use or construction materials is available for a limited subset of OSM buildings, but not for other data products so it was not considered in these analyses.

In the first method of quantifying counts of building footprints per settlement, predefined settlement extents were used – specifically the GRID3 v2.0 Settlement Extent dataset [58]. This dataset consists of vector polygons, delineating outlines of settlements or groups of buildings which have been created through geospatial processing of Ecopia Digitize Africa “year 2” building footprints. These settlement extents represent geographic groupings of building footprints, and therefore do not necessarily correspond to individual settlements recognised by local or national administrations or the communities residing there. Settlement extents are also not named. Consequently, a single named settlement may be represented in the GRID3 settlement extent dataset as multiple polygons, and conversely a single settlement extent polygon may represent multiple named settlements. The GRID3 v2.0 settlement extents include a settlement type attribute based on the Degree of Urbanisation approach [59]. We used this Level 1 Degree of Urbanisation attribute (rural, urban cluster or urban centre) to stratify settlement extents by rural/urban types.

### Data processing

**Fig. 1:**
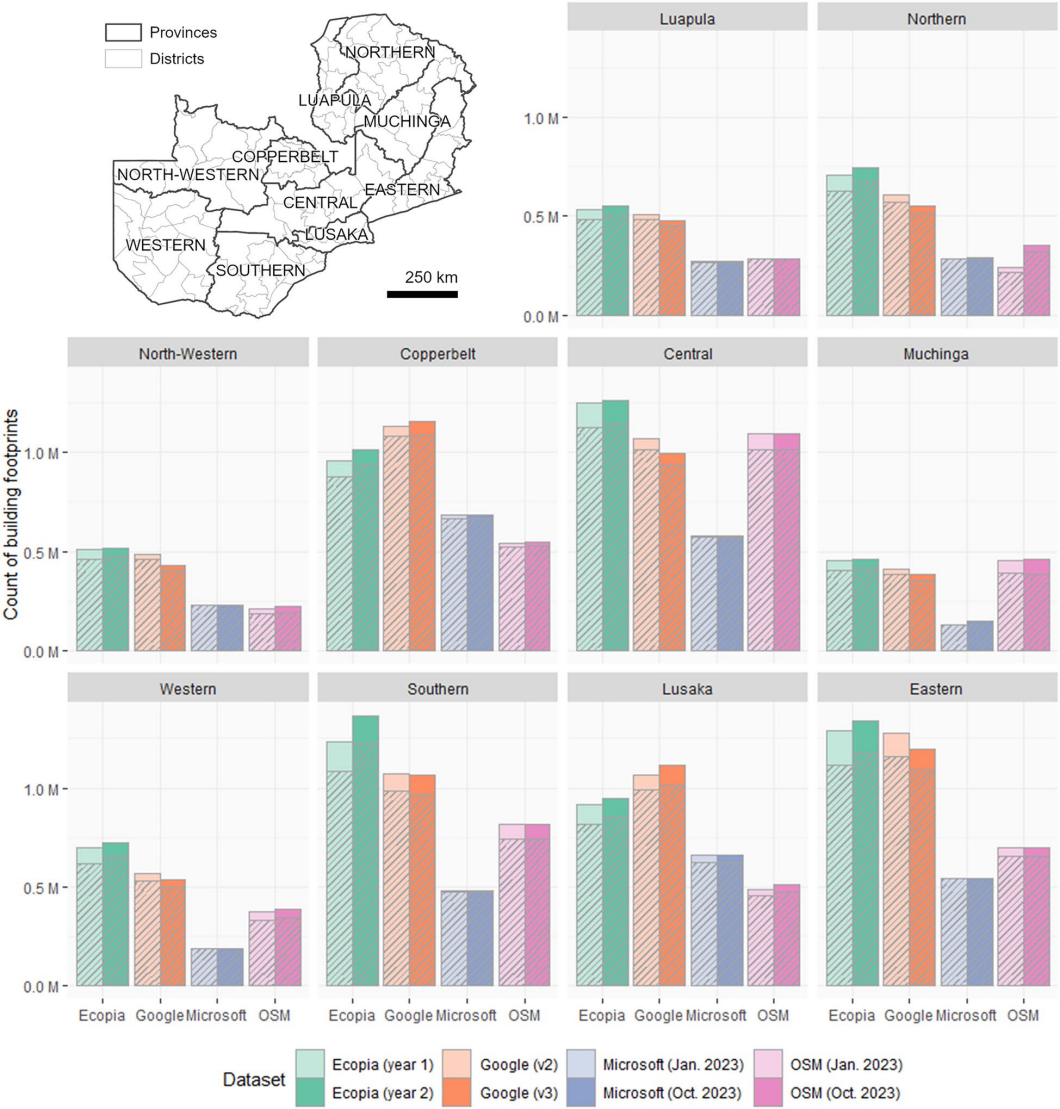
The count of building footprints per province, for each building footprint data product. Total counts are displayed as solid bars. The subset of “potentially residential” building footprints (≥ 9 m^2^ and ≤ 330 m^2^) are indicated by the striped fill

For each building footprint dataset, polygons were converted to a common geographic coordinate system (WGS84, EPSG:4326) and the geodesic area of each building footprint polygon was calculated in square metres. Any building footprint polygon with a geometric centroid outside the national boundary of Zambia was excluded. As IRS campaigns are specifically targeted at buildings in which residents sleep, only the subset of building footprints that were most likely to be residential, were included (henceforth referred to as “potentially residential”). As has been used previously in IRS campaigns in Zambia, minimum and maximum area thresholds were applied to exclude probable non-residential buildings [35]. Thus, for each dataset, building footprint polygons that were < 9 m^2^ (probable toilets or granaries) or > 330 m^2^ (likely non-residential structures such as churches, warehouses or commercial premises) in area, were excluded from the analyses (Fig. 1).

The first method of calculating counts of building footprints per settlement, utilised the GRID3 v2.0 settlement extents dataset as the basis for defining settlements. The GRID3 settlement extents are derived from “year 2” Ecopia building footprints, which have been buffered by 50 m and then overlapping buffers dissolved. Utilising these settlement extents with other sources of building footprints will likely result in some building footprints being located outside settlement extents, particularly in locations experiencing settlement growth. For this reason, we expanded the spatial coverage of each GRID3 settlement extent by up to 1 km, with any building footprints that were within 1 km of a settlement extent, included in the count of building footprints of the settlement extent that they were nearest to.

A second method was also tested, which did not utilise pre-defined settlement extents and instead, for each building footprint dataset in turn, groupings of neighbouring building footprints were created based on a proximity threshold. Recent IRS campaigns in Zambia have used this approach with a range of proximity thresholds, from 50 m [34] up to 250 m [46]. For these campaigns, proximity thresholds were decided based on both expected travel distances of mosquitoes and practical considerations around minimising travel time for IRS field teams, so as to maximise spraying of neighbouring structure and community-level protection. In this analysis, we utilised a proximity threshold of 100 m (in line with both previous IRS campaigns and the buffer distance used in deriving GRID3 v2.0 settlement extents), such that building footprints within 100 m of each other, were grouped into a cluster. For each building footprint dataset in turn, clusters were delineated by buffering all potentially residential building footprints by 50 m and dissolving all overlapping buffered areas. The resulting delineated clusters of building footprints are henceforth referred to as “derived settlement clusters”.

**Fig. 2:**
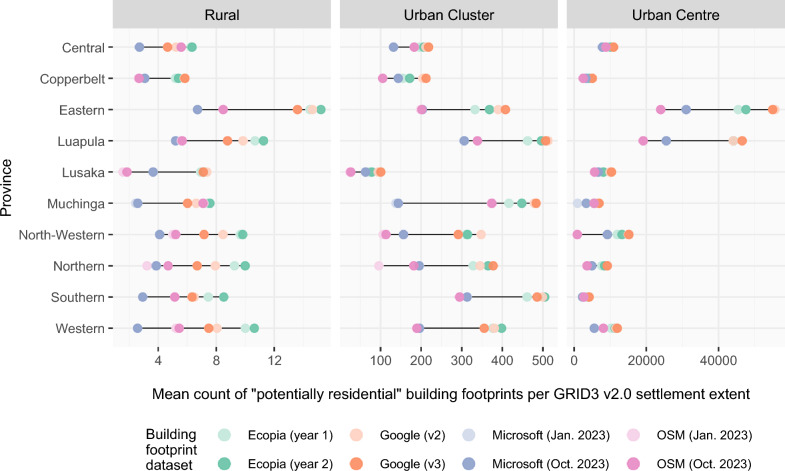
Mean counts of potentially residential building footprints per GRID3 v2.0 settlement extent, shown for each dataset and stratified by province and L1 degree of urbanisation

**Fig. 3:**
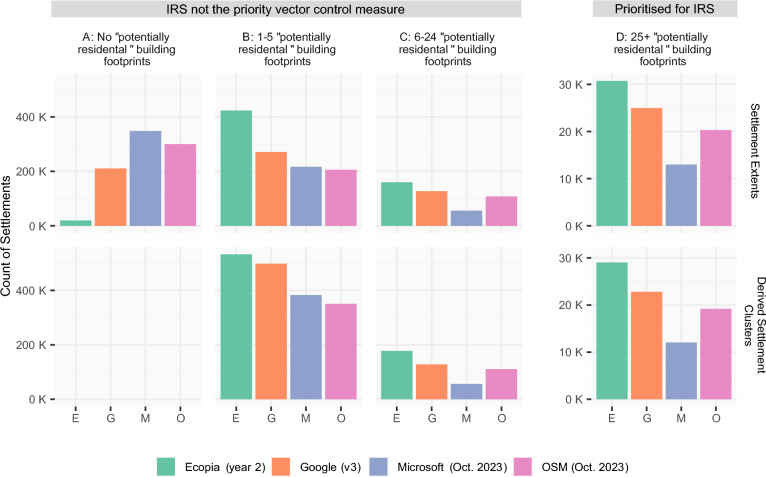
The count of settlements in classes A–D, based on the count of potentially residential building footprints per settlement (upper row: GRID3 v2.0 settlement extents, lower row: derived settlement clusters), for each building footprint dataset. Derived settlement clusters are formed based on the presence of building footprints and so no Class A settlements are produced with this method. Note that the y-axis values differ between classes A–C and class D

For both settlement definitions, the counts of potentially residential building footprints per settlement extent/derived settlement cluster were then calculated for each of the four building footprint data products (Figs. 2 and 3).

### Stratification of settlements

In identifying potential settlements suitable for IRS, settlements were classified based on threshold counts of potentially residential building footprints. Recent IRS campaigns have utilised a minimum threshold in terms of the count of potentially residential structures/buildings per settlement, with a threshold of 25 residential buildings commonly employed to ensure operational efficiency [34]. For IRS campaigns in Zambia, this threshold was initially applied based on groupings of buildings that were all located within a specified distance of each other (i.e. following the second definition of settlement described above). More recently, the same threshold has been applied to counts of potentially residential buildings within pre-defined GRID3 settlement extents [47]. In our analysis we have employed both definitions of settlement, with the same count threshold of 25 potentially residential building footprints per (i) expanded GRID3 settlement extent and (ii) derived settlement cluster, applied. For each building footprint dataset in turn and considering only the subset identified as potentially residential (≥ 9 m^2^ and ≤ 330 m^2^), settlements were stratified into four classes: no building footprints (class A), 1–5 building footprints (class B), 6–24 building footprints (class C) and 25 or more building footprints (class D, Fig. 3). From the stratified settlements, class D settlements were considered as the priority locations for IRS implementation, in line with criteria used in recent IRS campaigns in Zambia.

### Comparative analysis

To assess the impact of the choice of building footprint dataset on the identification of priority settlements for IRS campaigns, we calculated summary statistics related to the count, location and spatial similarity of class D settlements (those with 25 or more potentially residential building footprints). Our comparative analysis focussed on the most recent version of each of the four building footprint data products. The agreement in class D settlement extents across building footprint datasets was assessed in terms of the count (n = 1–4) and combination of datasets (Figs. 5 and 6 and Supplementary Figs. A1 and A2). For each building footprint dataset, the count of class D settlement extents per province (administrative unit level 1) with rural/urban stratification was calculated, along with the count of building footprints within these settlement extents (Fig. 7 and Supplementary Table A5).

To further assess geographic similarity in class D settlement extents, Jaccard coefficient values were calculated for each province, on a pairwise basis between datasets (Supplementary Fig. A3). The Jaccard coefficient (*J*(X,Y) =| X ∩ Y |/| X ∪ Y|) was calculated as the count of settlement extents that were identified as class D with two building footprint datasets (X and Y), divided by the count of class D settlement extents identified with building footprint dataset X and/or Y. This calculation was not weighted by the area of settlement, such that settlement extents with 25 buildings and settlement extents with, for example, 5000 buildings were both considered equally. For the subset of settlement extents classified as class D with all four building footprint datasets, statistics related to the variability in counts of building footprints per settlement extent were also calculated (Supplementary Tables A3, A4 and Supplementary Fig. A4).

## Results

Comparing counts of building footprints and potentially residential building footprints per province across Zambia (Fig. 1), shows considerable variation between data products (Ecopia, Google, Microsoft and OSM). For the majority of provinces, counts of building footprints were highest for Ecopia (eight provinces) or Google (two provinces—Lusaka and Copperbelt), and lowest for Microsoft (six provinces) or OSM (four provinces). For Central and Muchinga province however, the count of OSM building footprints is greater than the count of Google footprints.

For each building footprint data product, two datasets were included in this comparison of building footprint counts, shown as pairs of bars in Fig. 1. The two datasets are either the two most recent versions, or in the case of OSM and Microsoft, data extracted at two time points (January and October 2023). The comparison of building footprint counts per province, between pairs of datasets, show variations in counts, with patterns differing between provinces. Counts of Ecopia building footprints per province increased between the “year 1” and “year 2” datasets. Counts of Google building footprints increased between v2 and v3 for a couple of provinces (Copperbelt and Lusaka) but decreased for all other provinces. Counts of OSM and Microsoft building footprints per province showed little change between dataset versions, except for Northern province, where counts of OSM buildings increased considerably from 243,627 in January 2023 to 350,379 in October 2023.

When minimum (≥ 9 m^2^) and maximum (≤ 330 m^2^) area thresholds are applied, the proportion of the total building footprints that are excluded varies between provinces and data products (Fig. 1). For most provinces, the greatest reduction in count of building footprints is observed for Ecopia, with minimal change in the count of Microsoft building footprints, except for Copperbelt and Lusaka provinces (Fig. 1). Note that for the remaining analyses, only the subset of building footprints considered to be potentially residential (≥ 9 m^2^ and ≤ 330 m^2^) are included.

Calculating counts of potentially residential building footprints per settlement extent also shows considerable variation between building footprint data products. Figure 2 shows the mean count of potentially residential building footprints per settlement extent, stratified by province and Level 1 degree of urbanisation [59]. For rural settlement extents, the mean count of potentially residential building footprints per settlement is generally highest for Ecopia and lowest for Microsoft or OSM. In rural areas of Muchinga and Central provinces, mean counts for Ecopia, Google and OSM are very similar, with mean counts of Microsoft building footprints being lower. In both Urban Clusters and Urban Centres, Google has the highest mean count of potentially residential building footprints per settlement extent for most provinces, and OSM the lowest. For settlement extents classified as Urban Centres, the magnitude of difference in mean counts between building footprint datasets in some provinces is massive, for example in Eastern province, the mean count of potentially residential building footprints per settlement extent is 23,961 for OSM (Jan. 2023) and 54,940 for Google (v3). Similarly, for Luapula province, the mean count of potentially residential building footprints per Urban Centre extent ranges from 19,123 (OSM Jan. 2023) to 46,481 (Google v3). There is only a single settlement extent classified as an Urban centre in Eastern, Luapula, North-Western, Northern and Southern provinces, and thus the mean counts per Urban centre for these provinces, reflect values only from one settlement extent.

Counts of building footprints per settlement have been used in planning past IRS campaigns in Zambia to identify priority settlements. Figure 3 shows, for each building footprint dataset, the count of settlements in classes A-D, when settlements are classified based on the count of potentially residential building footprints per settlement (considering only the most recent version of each data product). Nationally, the count of settlements with 25 or more potentially residential building footprints (class D) is greatest when Ecopia year 2 building footprints are used (30,749 settlement extents and 29,032 derived settlement clusters). The second highest count of class D settlement is for Google v3 (24,977 settlement extents and 22,806 derived settlement clusters), followed by OSM (20,330 settlement extents and 19,202 derived clusters). The count of class D settlement extents based on Microsoft building footprints is the lowest of any data product (12,982 settlement extents and 12,043 derived clusters). Figure 3 also shows the breakdown of the remaining settlements when threshold counts of 0 (class A), 1–5 (class B) and 6–24 potentially residential building footprints (class C) are applied for each data product. Counts of class B and C settlement extents and derived clusters were highest for Ecopia year 2 building footprints. Conversely, the count of settlement extents with no potentially residential building footprints (class A) was highest for Microsoft. Counts of potentially residential building footprints within each settlement class are summarised in Supplementary Tables A1 and A2.

The count of settlements in each class nationally with different building footprint data products (Fig. 3) however hides any geographic variation in the classification of settlements, when different building footprint data products are used. The count of settlement extents in each class (the upper part of Fig. 3), is shown in Supplementary Fig. A1, with the addition of nested bars. These nested bars show the subset of identical settlement extents that are classified as the same class, with each building footprint dataset. For example, of the 12,982 class D settlement extents selected when a threshold of 25 is applied with Microsoft building footprints, 12,754 (Ecopia), 12,326 (Google) and 10,135 (OSM) of the same settlement extents are also classified as class D when the same threshold is applied with the other building footprint datasets. Of the 30,749 class D settlement extents with Ecopia building footprints, a subset of 21,922, 12,754 and 17,904 are class D when the same threshold is applied to Google, Microsoft and OSM building footprints respectively.

**Fig. 4:**
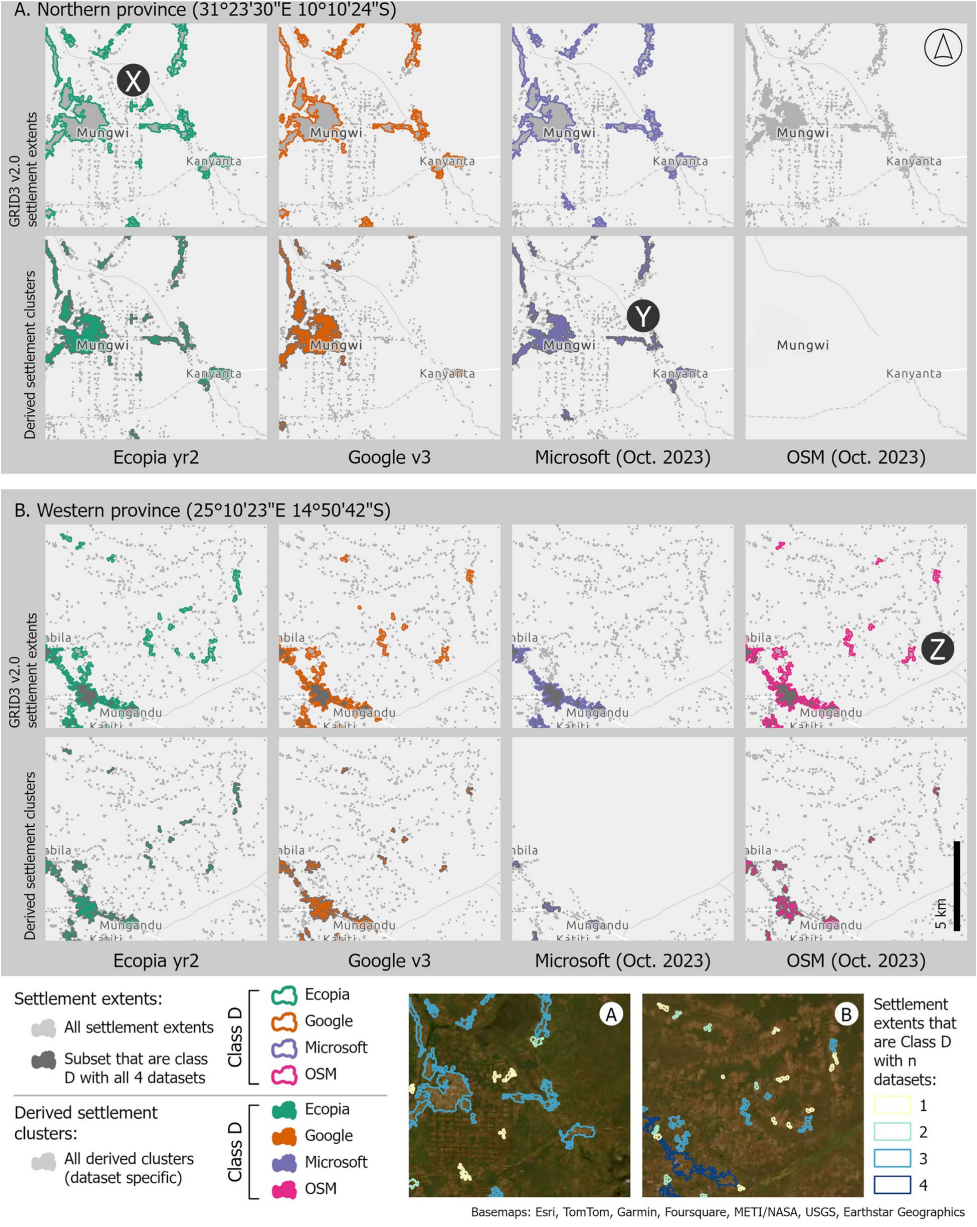
Mapped examples of class D settlement shown for two example locations (A and B). For these locations, the class D settlement extents (upper) and class D derived settlement clusters (lower) are shown for each building footprint dataset (left to right: Ecopia, Google, Microsoft and OSM). Three example locations with clear differences between datasets are marked as X, Y and Z

For some locations, class D settlements are quite similar between building footprint datasets, but for other locations the choice of building footprint dataset and/or the settlement definition has a considerable impact on which settlements meet the threshold for class D. In Fig. 4, the class D settlements are shown for both GRID3 settlement extents (top row of both panels) and derived settlement clusters (bottom row of both panels), for two example locations. The location marked X shows class D settlement extents (and derived settlement clusters) with Ecopia, but no class D settlement extents for the other three datasets. Location Y has class D derived clusters with Microsoft and Ecopia only, but class D settlement extents are identified in the same location with Microsoft, Ecopia and Google. Location Z has class D settlement extents with OSM, Google and Ecopia, but no class D derived clusters for OSM, and much smaller clusters for Google and Ecopia. In addition, the settlement definition can have considerable impact on which settlements are identified as class D (see for example the differences between class D settlement extents and derived clusters with Google v3 in panel (a) and Microsoft in panel (b) of Fig. 4).

**Fig. 5:**
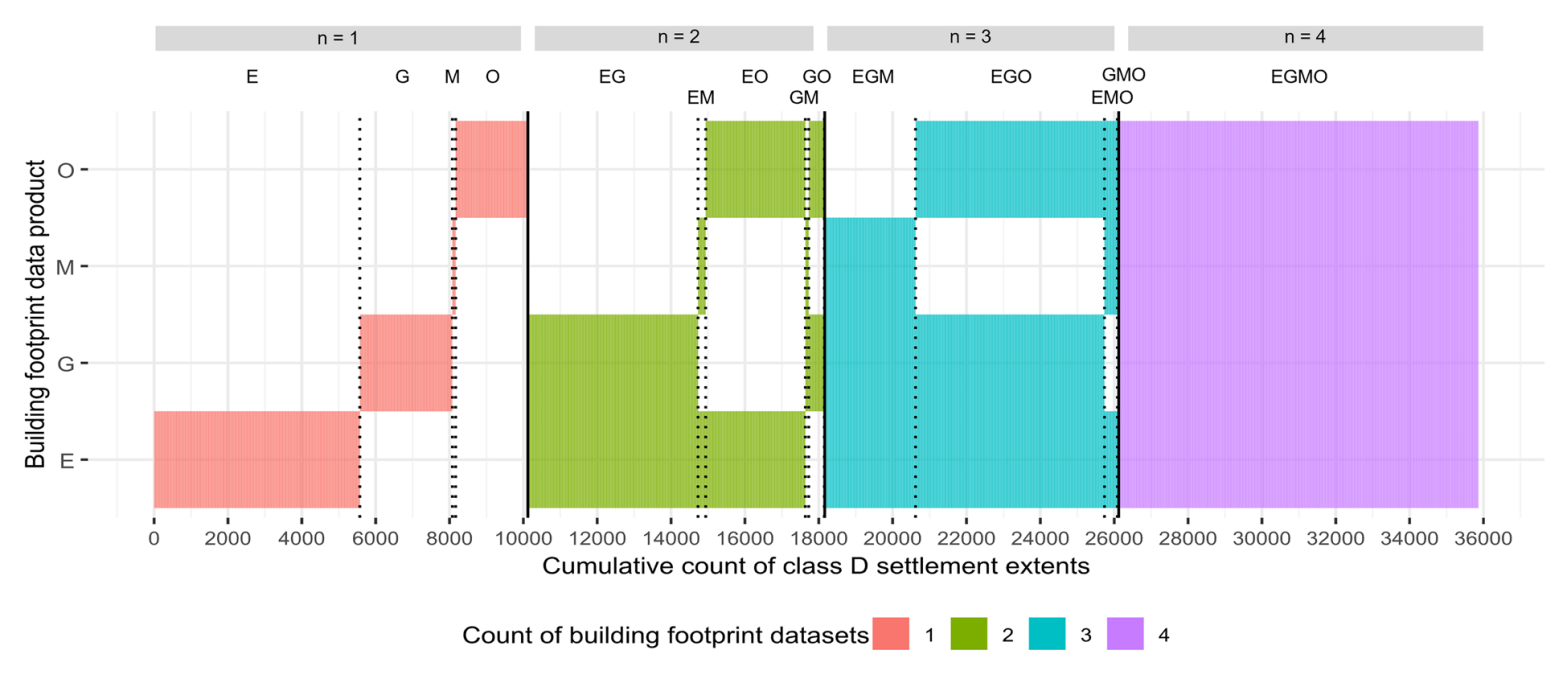
Cumulative count of Class D settlement extents stratified by the count of building footprint datasets in agreement (n = 1–4). Within each stratum (X-axis), the total count of class D settlements is labelled by the combination of building footprint datasets (E = Ecopia year 2, G = Google v3, M = Microsoft (Oct. 2023 download) and O = OSM (Oct. 2023 download))

Figure 5 further explores the dissimilarity in settlement extents classified as class D with each building footprint dataset. In total across the four datasets, 35,861 settlement extents were identified as class D (IRS priority settlements) as they had 25 or more potentially residential building footprints in one or more building footprint datasets. Of these, there were 9738 settlement extents identified with all four building footprint datasets (EGMO). Conversely there were 10,123 settlement extents identified in only one (E, G, M or O) of the four building footprint datasets, with over half of those settlement extents only being identified in terms of counts of Ecopia building footprints (n = 5571). A smaller number of settlement extents were only selected with Google (n = 2506) and OSM (n = 1957), with a minimal number selected only with Microsoft (n = 89). For settlement extents classified as class D with two or three datasets, the majority included combinations of Ecopia with Google and/or OSM (e.g. EG, EO, EGM and EGO). Supplementary Fig. A2 shows the locations of the selected settlement extents for each building footprint dataset combination.

**Fig. 6:**
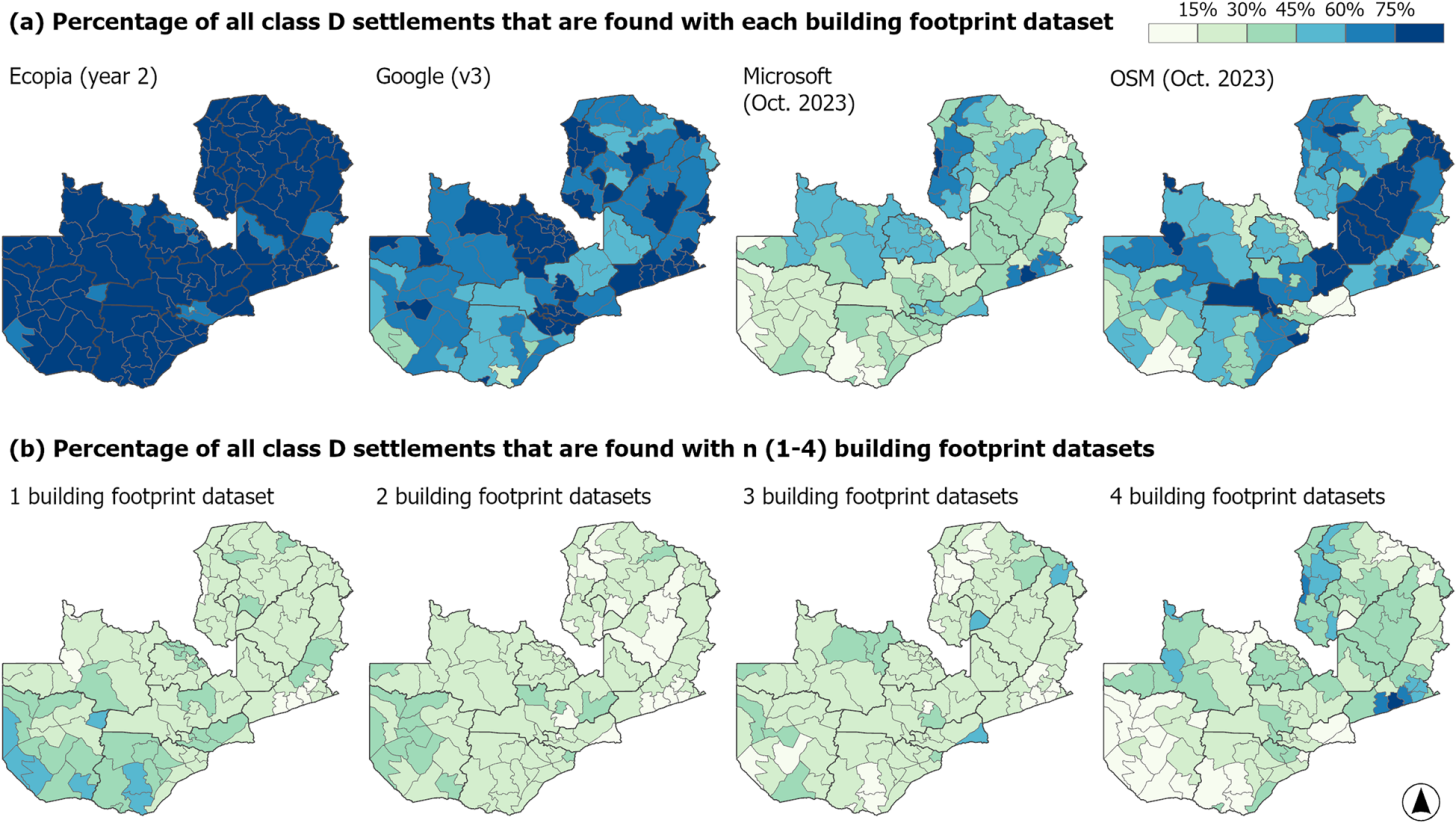
For all settlement extents classified as class D with one or more building footprint datasets (n = 35,861), **a** the percentage that are classified as class D with each dataset, and **b** the percentage classified as class D with 1–4 datasets, is mapped at district level

Considering all settlement extents classified as class D with one or more building footprint datasets, the percentage classified as class D with each building footprint dataset is mapped at district level (Fig. 6a). The percentage of class D settlement extents found with each building footprint varies considerably, with Ecopia having the most districts (n = 102/116) with over 75% of all class D settlement extents selected. In contrast, Microsoft had the fewest districts with over 75% of all class D settlement extents selected (n = 2). With Google and OSM, more spatial variability in the percentage of class D settlements with each dataset, is observed (Fig. 6a). In Fig. 6b, the percentage of settlement extents classified as class D in only one dataset, and in two, three or four datasets is mapped per district. The proportion of settlement extents classified as class D with just one dataset is greatest in selected districts in Southern and Western provinces, with seven districts having over 45% of class D settlement extents classified as such with just one building footprint dataset. Conversely, four districts in Eastern province, two districts in North-Western and one district in Luapula had less than 15% of class D settlement extents as class D with just one building footprint dataset. These same districts all had more than 45% of class D settlement extents classified as such with four buildings footprint datasets, along with six districts in Luapula and one in Northern. Sinda district in Eastern province had the highest proportion of class D settlement extents classified as such with all four building footprint datasets (82%).

The spatial dissimilarity of class D settlement extents is further explored in Supplementary Fig. A3, with Jaccard Coefficient values for each pairwise combination of building footprint datasets, stratified by province. In terms of class D settlement extents, the highest Jaccard Coefficient values (greatest spatial similarity in settlement extents) are found with Google and Ecopia building footprints for all provinces apart from Muchinga and Central (Supplementary Fig. A3). For Muchinga and Central provinces, the spatial similarity between class D settlement extents is greatest between Ecopia and OSM. There is considerable variation in Jaccard Coefficient values between provinces, with Luapula and Eastern provinces tending to have higher values, and Western province lower values, for most pairwise combinations. The same pairwise combination of datasets can vary a lot between provinces, for example OSM and Microsoft has a Jaccard coefficient of 0.66 for Luapula, but only 0.24 for Western province.

**Fig. 7:**
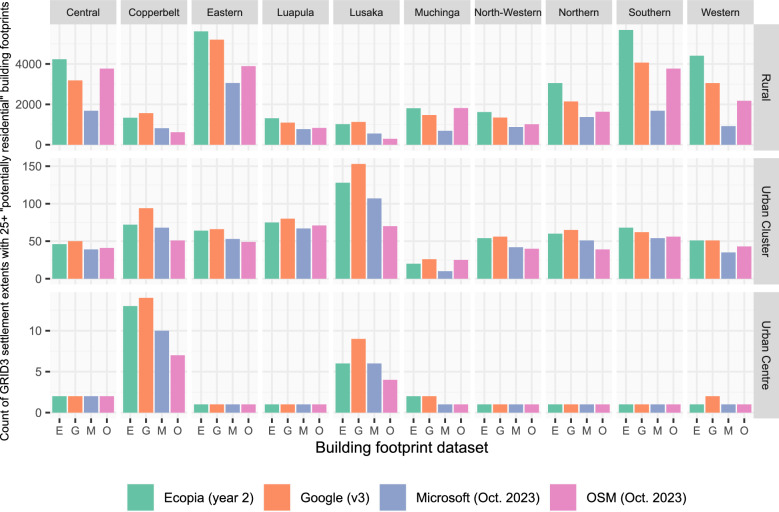
The count of class D settlement extents selected with each building footprint dataset, stratified by province, and the L1 degree of urbanisation class. Note that the y-axis values vary between the L1 degree of urbanisation strata

In Fig. 7, the count of class D settlement extents for each building footprint dataset is stratified by province and settlement type. In rural areas, the count of class D settlement extents is highest based on Ecopia building footprints in 8 provinces, with the count of class D settlement extents based on Google building footprints being higher in two provinces (Lusaka and Copperbelt). Across all provinces, the number of rural class D settlement extents with Microsoft building footprints is considerably less than the number of class D settlement extents with Ecopia building footprints; the count of Microsoft rural class D settlement extents being less than 65% of the count of Ecopia rural class D settlement extents, and as low as 21% in Western province. In some rural settings, the number of OSM class D settlement extents is comparable to Ecopia or Google (e.g. Central and Southern), but in other provinces (e.g. Lusaka) there are far fewer OSM class D settlement extents.

In urban clusters and centres, the total number of class D settlement extents is expectedly fewer (although the extents themselves are larger) and there is also less variation in the total number between building footprint datasets. For Eastern, Luapula, North-Western, Northern and Southern provinces, there is only a single class D settlement extent classified as an urban centre (Fig. 7). For urban centres in most other provinces, the count of class D settlement extents is highest for Google building footprints. Even with very similar counts of class D settlement extents with different building footprint datasets, there is considerable variation in the total count of building footprints within class D settlement extents (Supplementary Table A5). In the most extreme example, within class D urban centre settlement extents in North-Western province, there are 92,739 Ecopia, 106,216 Google, 64,280 Microsoft and only 6,123 OSM building footprints. Considering just the subset of settlement extents that are classified as class D with all four building footprint datasets (n = 9738, labelled as EGMO in Fig. 5), there is still major variability in counts of building footprints per settlement extent (Supplementary Tables A3/A4 and Supplementary Fig. A4).

## Discussion

High-resolution geospatial data on population, settlements and infrastructure are increasingly being utilised for planning and implementing public health interventions, including as part of malaria control programmes. For vector control measures to be effective in reducing malaria transmission, high- and uniform-coverage of communities is needed [41, 60]. Here we focus on a case study of planning for IRS campaigns in Zambia, where new geospatial datasets and technology have been integrated over the past decade. We show that if settlements are classified by count and proximity of buildings, the settlements identified for potential inclusion in IRS campaigns differ considerably depending on the choice of building footprint dataset.

### Relevance and practical implications of findings

The results of our analysis show that both the overall count and the geographic location of class D settlements (those settlements with at least 25 potentially residential building footprints), varies notably between building footprint datasets. Focussing on the most recent version of each of the four building footprint datasets, the count of class D settlement extents nationally varied between 12,982 and 30,749, when Microsoft (October 2023 version) and Ecopia year 2 building footprint datasets were used respectively (Fig. 3). Considering all class D settlement extents identified with any of the four building footprint datasets, the percentage of settlements identified as class D with each dataset in turn also shows considerable geographic variation between datasets (Fig. 6). When stratified by province (administrative unit level 1), the count of class D settlements in rural clusters was typically highest with Ecopia building footprints, and lowest with Microsoft building footprints (Fig. 7), at least in part likely reflecting gaps in data coverage (Fig. 4). Whilst in urban clusters, the count of class D settlements was highest with Google building footprints in most provinces, and lowest with either OSM or Microsoft buildings footprints.

Given the geographic differences observed in class D settlements with different building footprint datasets, these results highlight how the choice of building footprint dataset may bias the selection of settlements (and consequently population groups). Selecting a particular building footprint dataset may result in locations with certain characteristics (e.g. predominantly urban areas) being potentially prioritised or conversely excluded. For example, using Microsoft building footprints to identify rural class D settlements results in far fewer rural class D settlements, than if counts of buildings are based on Google or Ecopia building footprints (Fig. 7). Such differences could introduce inequalities in campaign provision, as recently also found by Gevaert et al. [50]. Although not the primary focus of this study, the settlement definition (GRID3 settlement extents or derived settlement clusters) used as the basis for calculating counts of building footprints per settlement, also affected the number and geographic location of settlements selected (Figs. 3 and 4). Whilst settlement and building footprint data are not the sole data used in decision making concerning malaria vector control initiatives, our results show that the use of a particular dataset has the potential to influence decisions and strategies.

The WHO guidance for IRS does not specify an absolute coverage target, acknowledging that 100% coverage is rarely possible, but a minimum spray target of 80% of eligible structures is commonly used [61]. In this context, an accurate count of geo-located residential buildings is necessary for (i) planning to ensure that available resources are targeted to appropriate locations to maximise reductions in malaria transmission, (ii) ensuring that sufficient spray teams, equipment and insecticide are allocated and delivered to the right locations, and (iii) to assess spray coverage after campaign completion. The results of our analysis quantify the relative differences in counts of building footprints per settlement (Figs. 2, 7 and Supplementary Fig. A4) and highlight the sensitivity of these metrics to the choice of building footprint dataset (Supplementary Tables A3 and A4). For example, if the subset of settlement extents that are classified as class D with all four building footprint datasets were all identified as appropriate locations for IRS, for Western province this would mean planning and implementing IRS in 532 settlements (Supplementary Table A4). For these selected settlements, the choice of building footprint dataset still would have a big impact on the estimation of required resources and assessment of spray coverage. Considering a hypothetical IRS campaign, in which 50,000 buildings were sprayed in Western province, coverage rates across all target settlements in Western province would be calculated as 60.4% (out of 83,803 Ecopia building footprints), 74.4% (out of 67,225 Google building footprints), 129.8% (out of 38,531 Microsoft building footprints) or 82.9% (out of 60,326 OSM building footprints).

The detailed nature of building footprint polygon data can give an illusion of accuracy, more so than, for example, raster land cover classification maps. Building footprint datasets can commonly have some buildings omitted (false negatives) and other features misclassified as buildings and thus included (false positives) [62]. These can occur due to gaps in satellite imagery coverage, satellite imagery being outdated, limitations of the feature extraction algorithm, or buildings being obscured in imagery due to tree canopy cover, for example. A lack of metadata can further compound these problems, as the definition of what is considered as an individual building, details of the feature extraction algorithms and post-processing of extracted features, and the temporal imagery coverage, can all vary between data products [31]. As building footprint datasets evolve and are used in different fields, there is a need for data producers to take steps to address these issues and support data users. Those working with building footprint data to support decision making, in the context of health programme planning and more widely, also need to be aware of differences between data products, to consider potential impacts of choosing a particular dataset and to be cognisant of their limitations. As of October 2023, in addition to buildings from OpenStreetMap, there were at least three building footprint data products in existence for Zambia, with two or more versions of each dataset (Fig. 1). Apart from Ecopia building footprints, no other datasets include attribute information on the date of source satellite imagery, making it impossible to know the timepoints that building footprints are representative of.

### Recommendations

From the building footprint data products included in our analysis, we would suggest that generally the dataset coverage is most complete for Ecopia and Google building footprints. However, this recommendation has several caveats: our comparative analysis does not include an authoritative reference dataset, the Ecopia building footprints are based on satellite imagery from 2020 or earlier (over half of building footprints were extracted from satellite imagery collected in 2019) and no granular information is provided on source imagery dates for Google. In locations where there have been recent, comprehensive campaigns to digitise buildings in OSM, these data may be more comprehensive in their coverage and better reflect the structure of settlements, but this is difficult to assess at scale. Recent work indicates a likely move towards more dynamic updates of building footprints and settlement datasets in the future [63, 64], as well as potential improvements in building height and typology estimation [26, 65]. In the context of health programme planning, these developments would likely be beneficial in improving estimation of population numbers [66] and hence programme denominators, as well as enabling settlement change detection with temporally-explicit building/settlement datasets. For IRS campaigns in particular, such data may help in identifying residential buildings, and potentially guide in the estimation of required insecticide spray volumes.

This study has considered the use of building footprint data products in isolation, but with a growing number and availability of data products, the potential to use data products in combination increases. Further work is needed to develop systematic and robust methods and guidelines to do this. Opportunities to efficiently conduct field validation of satellite-derived datasets, as part of field-deployed health campaigns, should also be pursued. The results of field validation can be used to further improve building datasets, ultimately accelerating planning and delivery of health interventions.

Although building footprint datasets extracted from satellite imagery provide new opportunities to support geo-enabled microplanning at scale for a range of health programmes they are not a replacement for local knowledge or comprehensive field data collection. Enumeration of populations, households and/or buildings may be collected by health facilities through community-based service delivery campaigns and for headcount registers, but these are often not up to date, complete or accurate due to resources and capacity limitations [47, 48, 67, 68]. Geospatial data on counts and locations of buildings that have been field-verified (for example during resourced campaigns), likely provide the most accurate data on residential buildings for resource planning and delivery [69]. This is especially true if field service delivery teams use maps to navigate to all mapped building footprints, then verify that a building exists, add any unmapped buildings and label whether a building is residential or not. Field-verification of this sort has been conducted in Zambia using the Reveal platform mobile app which enables in situ updating of building data and denominators for each implementation cycle [36]. However, this data is usually limited to selected campaign prioritised areas, and therefore not available to all health facilities and districts—making it challenging, but not impossible, to use at scale. In Zambia, building footprints extracted from satellite imagery and field-verified data have been used in combination to inform resource needs during campaign planning and to monitor delivery [40, 69]. It is crucial that decision makers still critically assess any such data against existing sources, such as those available at health facility level, in order to ensure that service delivery targets and denominators are as accurate as possible.

### Limitations

Aside from IRS planning in Zambia, building footprints or similar datasets have been used for geo-enabled microplanning in a range of other public health interventions (e.g. [31, 67, 70], with no data available for some datasets in some areas (for example, conflict-affected locations have been excluded from Google Open Buildings datasets) and a rapidly evolving data landscape.

Our analysis has several caveats. In identifying potential priority locations for IRS, we applied a set of criteria concerning area of, distance between and counts of building footprints. As the national strategy in Zambia has changed over time, the criteria and guidelines used for planning vector control campaigns have similarly evolved [51, 71]. The criteria used in our comparative analysis are based on campaigns implemented with the NMEP over the past decade, and provide a framework within which to assess the impact of utilising different building footprint datasets for IRS planning. Our analysis has considered solely building footprint data to identify the subset of settlements that might be considered as suitable for IRS, and has not incorporated the important contextual knowledge of district teams to select the final settlements for inclusion in an IRS campaign.

In addition, none of the feature-extracted building footprint datasets (Google, Ecopia and Microsoft) included attributes on building type or use, necessitating an approximation of potentially residential buildings based on simple area thresholds (as has been used in previous work e.g. [35]). The subset of building footprints considered to be potentially residential based on simple area thresholds, will inevitably include some non-residential structures such as churches, shops or health facilities. Further work should explore more sophisticated classification approaches to identify non-residential/non-sprayable structures (e.g. [72, 73]), and assess the sensitivity of such classification approaches to different building footprint products. Given the lack of an authoritative national reference dataset, our analysis has been limited to comparisons between available building footprint datasets. As new sources of data become available, for example geolocated household data from the 2020 round of national population and housing censuses, these should be included in similar comparative analyses.

## Conclusions

The recent growth in the global availability of detailed building footprint data has provided new opportunities for integrating geo-enabled microplanning into public health interventions, particularly in previously data-scarce locations. This proliferation of datasets however also brings challenges for data users and decision-makers. The results of this study show that the choice of building footprint dataset has a substantial impact on locations selected when criteria are based on counts and proximity of buildings. This has been explored in the context of planning IRS campaigns in Zambia, but similarly large differences would be expected if count-based criteria are used for planning and evaluating other resource provision campaigns. Further work is needed to quantify the sensitivity of the choice of building footprint dataset in such analyses across a range of geographic contexts. Data producers could also better support data users through improved metadata, by routinely disclosing information on spatiotemporal coverage of datasets, definitions of features considered to be buildings and rates of omission and commission. As the building footprint and settlement data landscape continues to grow and diversify, understanding the characteristics and comparability of datasets will remain fundamental to robust data use. Caution will continue to be needed in selecting building footprint data products to integrate into analyses, and where possible, any conclusions drawn should be supported by local, contextual knowledge and field-verified data.

## Supplementary Information

Additional file 1.

## Data Availability

Google v3 building footprints are available to download in tiled format (level 4 S2 cells) from: https://sites.research.google/open-buildings/#open-buildings-download, or accessed via Google Earth Engine: https://developers.google.com/earth-engine/datasets/catalog/GOOGLE_Research_open-buildings_v3_polygons. OpenStreetMap building footprints can be extracted using various plugins, or pre-prepared national files updated daily are available from GeoFabrik: https://download.geofabrik.de/africa/zambia.html. Microsoft “global” building footprints are available to download from: https://github.com/microsoft/GlobalMLBuildingFootprints. Ecopia building footprints are not publicly available, but access can be requested for humanitarian purposes from Ecopia (https://www.ecopiatech.com/). The GRID3 v2.0 settlement extent dataset is available from: https://doi.org/10.7916/wqmn-f746.

## Abbreviations

IRS: Indoor residual spraying
LLIN: Long-lasting insecticide-treated net
NMEC: National Malaria Elimination Centre
NMESP: National Malaria Elimination Sstrategic Plan
OSM: OpenStreetMap
UNICEF: United Nations Children’s Fund
WHO: World Health Organisation
COVID-19: Coronavirus disease 2019, caused by the SARS-CoV-2 virus
