# Supplementary material for: Assessing the impact of building footprint dataset choice for health programme planning: a case study of indoor residual spraying (IRS) in Zambia
Source: Int J Health Geogr. 2025 May 24;24:13. doi: 10.1186/s12942-025-00398-7 (PMC12103797; doi:10.1186/s12942-025-00398-7)
Supplement: Supplementary file 1 — Additional file 1. [file 12942_2025_398_MOESM1_ESM.pdf]

Supplementary Information

For manuscript –

Assessing the impact of building footprint dataset choice for health programme planning: a case study of indoor residual spraying (IRS) in Zambia

Appendix A

	**Settlement Class (count of potentially residential building footprints per GRID3 v2.0 settlement extent)**				
	**A (n = 0)**	**B (n = 1-5)**	**C (n = 6-24)**	**D (n = 25+)**	**Total**
Counts of settlement extents					
Ecopia (year 2)	19,588	423,913	160,249	30,749	634,499
Google (v3)	210,573	271,318	127,631	24,977	634,499
Microsoft (Oct. 2023)	348,627	217,171	55,719	12,982	634,499
OSM (Oct. 2023)	300,276	206,096	107,797	20,330	634,499
Counts of potentially residential building footprints within settlement extents					
Ecopia (year 2)	0	978,965	1,662,155	5,438,017	8,079,137
Google (v3)	0	702,208	1,316,990	5,271,951	7,291,149
Microsoft (Oct. 2023)	0	501,561	564,180	2,931,462	3,997,203
OSM (Oct. 2023)	0	552,157	1,115,177	3,267,050	4,934,384

Table A.1: Counts of GRID3 settlement extents per class (A-D) and potentially residential building footprints within each settlement extent, for each building footprint dataset nationally.

	**Settlement Class (count of potentially residential building footprints per derived settlement cluster)**				
	**A (n = 0)**	**B (n = 1-5)**	**C (n = 6-24)**	**D (n = 25+)**	**Total**
Counts of derived settlement clusters					
Ecopia (year 2)	0	532,423	177,594	29,032	739,049
Google (v3)	0	497,970	127,966	22,806	648,742
Microsoft (Oct. 2023)	0	383,107	56,366	12,043	451,516
OSM (Oct. 2023)	0	350,556	111,138	19,202	480,896
Counts of potentially residential building footprints within derived settlement clusters					
Ecopia (year 2)	0	1,217,022	1,800,981	5,061,190	8,079,193
Google (v3)	0	1,085,408	1,288,624	4,955,366	7,329,398
Microsoft (Oct. 2023)	0	746,859	561,901	2,774,801	4,083,561
OSM (Oct. 2023)	0	821,479	1,122,911	3,002,920	4,947,310

Table A.2: Counts of derived settlement clusters per class (A-D) and potentially residential building footprints within each derived cluster, for each building footprint dataset nationally.

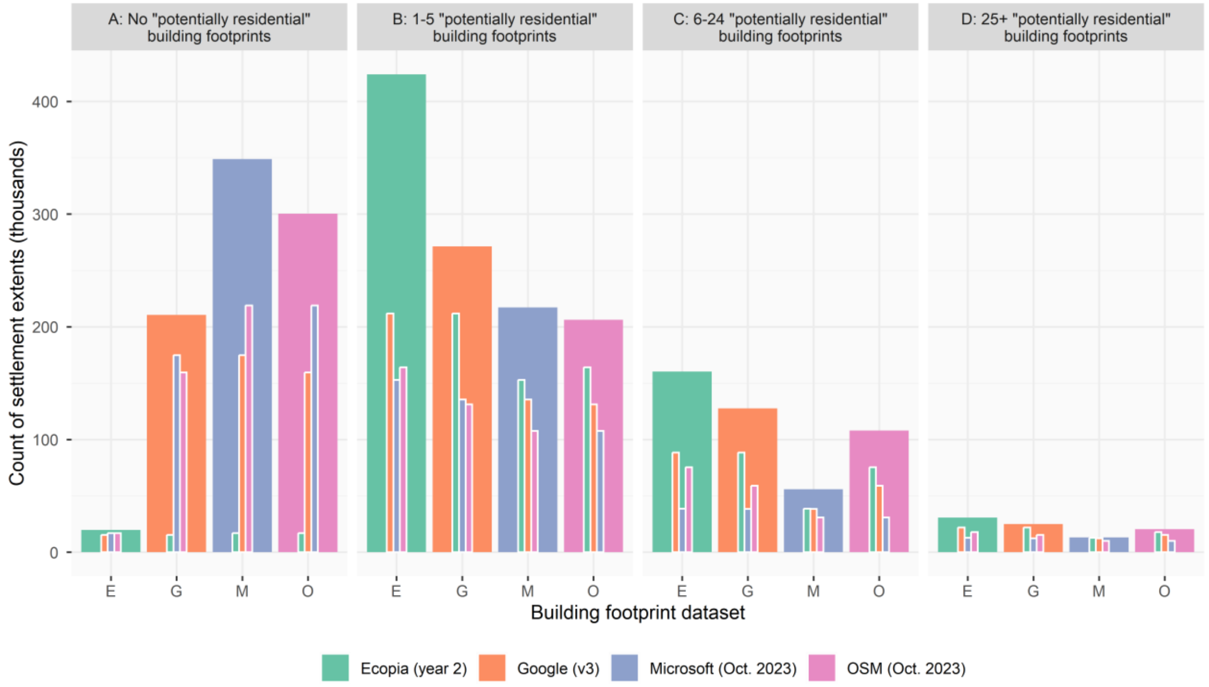

Figure A.1: The wider bars show count of GRID3 v2.0 settlement extents in each class (A-D), for each building footprint dataset, based on the count of potentially residential building footprints per settlement extent. For example, there are 12,982 class D settlement extents, when Microsoft building footprints are used to calculate the number of potentially residential building footprints per settlement extent. Within the wider bars, there are three smaller, nested bars, which represent the subset of settlement extents that are in the same class, when the three other building footprint datasets are separately used as the basis for calculating counts of potentially residential building footprints per settlement extent. For example, of the 12,982 settlement extents classified as class D with Microsoft building footprints, 12,754 are also classified as class D with Ecopia footprints, 12,326 are classified as class D with Google footprints and 10,135 are classified as class D with OSM footprints.

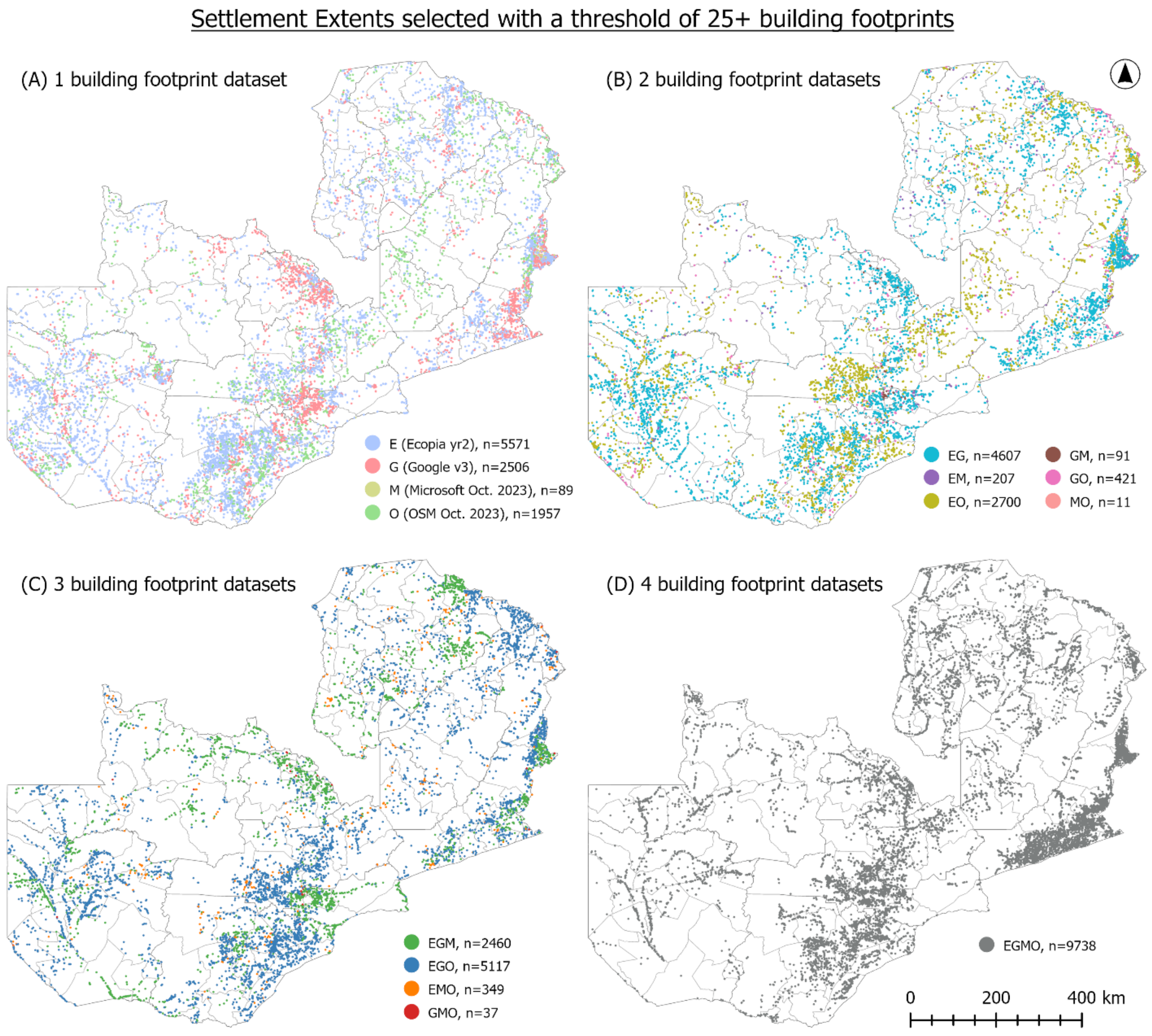

Figure A.2: The location of class D settlement extents (with 25 or more potentially residential building footprints), stratified by the count (A-D) and combination (colour of points) of building footprint datasets (E=Ecopia year 2, G=Google v3, M=Microsoft (Oct. 2023 download) and O=OSM (Oct. 2023 download)). Settlement extents are mapped based on their geometric centroid. The count of settlement extents is summarised in Figure 5.

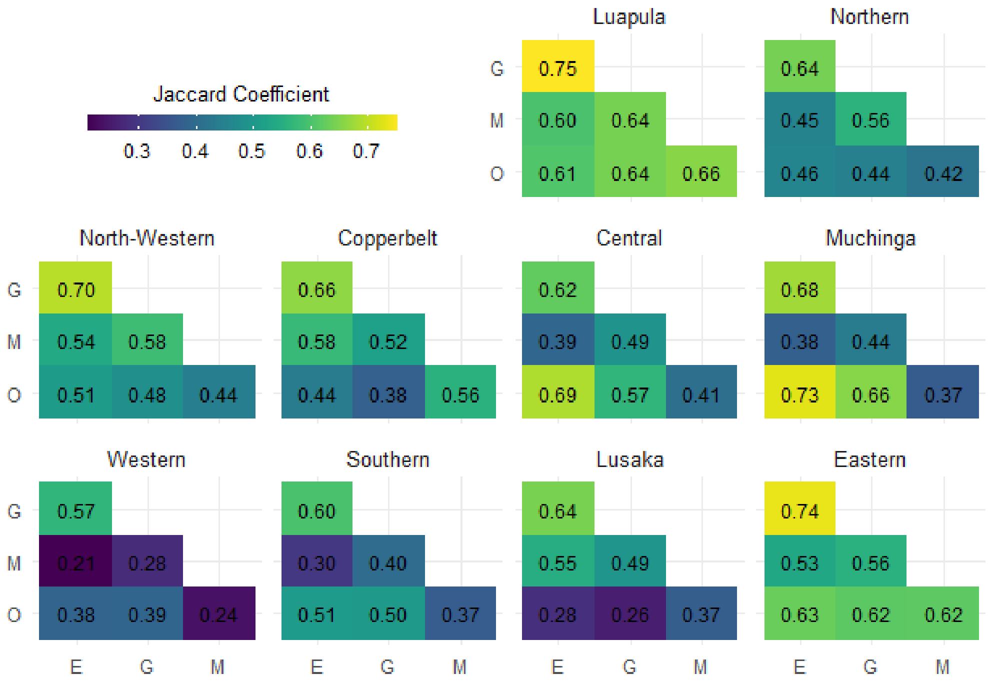

Figure A.3: Jaccard Coefficient values for pairwise comparison of the priority settlement extents selected with a threshold of 25 or more potentially residential building footprints, with different building footprint datasets (E=Ecopia year 2, G=Google v3, M=Microsoft (Oct. 2023 download) and O=OSM (Oct. 2023 download)), summarised for each province. Higher Jaccard coefficient values indicate greater spatial similarity in terms of the settlement extents selected.

Province	Count of class D settlement extents (EGMO)	Count of potentially residential building footprints within subset of class D (EGMO) settlement extents					
		Ecopia (year 2)	Google (v3)	Microsoft (Oct. 2023)	OSM (Oct. 2023)	Mean CV [%]	Median CV [%]
Central	1,515	381,485	371,125	248,527	342,897	46.7	19.0
Copperbelt	546	696,071	827,306	542,011	413,928	299.6	26.6
Eastern	2,651	736,852	717,235	395,036	439,896	71.3	37.3
Luapula	664	371,895	357,617	227,748	235,776	117.4	31.3
Lusaka	277	710,246	897,528	575,610	474,142	590.5	30.0
Muchinga	653	168,877	158,503	87,633	154,748	60.2	21.8
North-Western	573	284,371	262,940	168,300	124,429	162.6	33.5
Northern	845	285,925	257,101	157,935	180,749	82.3	28.1
Southern	1,447	420,340	386,769	233,169	284,998	65.4	20.5
Western	567	200,842	179,896	96,684	127,226	89.9	25.4
TOTAL:	*9,738*	*4,256,904*	*4,416,020*	*2,732,653*	*2,778,789*		

Table A.3: For the subset of class D settlement extents that are found with all four building footprint datasets (EGMO), the count of potentially residential building footprints within these settlement extents is shown per province. The mean and median coefficient of variation (CV) in the count of building footprints per settlement extent is also calculated for this subset of class D settlement extents.

Province	Count of class D settlement extents (EGMO)	Count of potentially residential building footprints within subset of class D (EGMO) settlement extents					
		Ecopia (year 2)	Google (v3)	Microsoft (Oct. 2023)	OSM (Oct. 2023)	Mean CV [%]	Median CV [%]
**Rural**							
Central	1,480	196,934	174,323	115,990	181,568	28.5	18.7
Copperbelt	491	93,476	107,125	67,165	51,946	50.1	24.6
Eastern	2,604	598,216	561,446	313,535	365,208	58.6	36.6
Luapula	596	153,245	132,615	92,678	96,798	50.9	27.6
Lusaka	206	40,532	44,247	24,975	19,774	58.4	25.1
Muchinga	642	109,364	89,107	58,712	102,139	37.3	21.8
North-Western	538	113,618	85,533	61,414	87,089	57.0	32.3
Northern	807	154,629	118,786	85,485	113,225	43.1	27.4
Southern	1,399	229,067	198,376	114,296	168,511	39.1	20.3
Western	532	82,803	67,225	38,531	60,326	38.5	24.0
TOTAL:	*9,295*	*1,771,884*	*1,578,783*	*972,781*	*1,246,584*		
**Urban Cluster**							
Central	33	72,926	76,829	46,255	64,653	469.3	61.9
Copperbelt	48	108,978	133,654	91,361	68,606	528.7	152.2
Eastern	46	91,085	100,849	50,501	50,714	522.3	71.5
Luapula	67	174,590	178,521	109,563	119,856	535.9	194.6
Lusaka	67	45,100	57,024	36,091	16,612	237.6	53.7
Muchinga	10	36,875	41,745	15,520	30,491	1,026.9	204.1
North-Western	34	78,014	71,191	42,606	31,217	707.3	245.5
Northern	37	80,765	83,489	43,019	45,892	561.8	94.9
Southern	47	160,205	155,211	100,299	95,019	717.7	69.6
Western	34	72,759	64,892	35,912	34,551	594.2	143.4
TOTAL:	*423*	*921,297*	*963,405*	*571,127*	*557,611*		
**Urban Centre**							
Central	2	111,625	119,973	86,282	96,676	6,557.1	6,557.1
Copperbelt	7	493,617	586,527	383,485	293,376	16,229.1	9,587.9
Eastern	1	47,551	54,940	31,000	23,974	12,414.2	12,414.2
Luapula	1	44,060	46,481	25,507	19,122	11,729.2	11,729.2
Lusaka	4	624,614	796,257	514,544	437,756	33,904.8	5,023.8
Muchinga	1	22,638	27,651	13,401	22,118	5,125.8	5,125.8
North-Western	1	92,739	106,216	64,280	6,123	38,449.1	38,449.1
Northern	1	50,531	54,826	29,431	21,632	13,933.7	13,933.7
Southern	1	31,068	33,182	18,574	21,468	6,183.2	6,183.2
Western	1	45,280	47,779	22,241	32,349	10,297.7	10,297.7
TOTAL:	*20*	*1,563,723*	*1,873,832*	*1,188,745*	*974,594*		

Table A.4: For the subset of class D settlement extents that are found with all four building footprint datasets (EGMO), the count of potentially residential building footprints within these settlement extents is shown per province, with rural/urban stratification. The mean and median coefficient of variation (CV) in the count of building footprints per settlement extent is also calculated for this subset of class D settlement extents. Rural/urban stratification is based on the GRID3 v2.0 settlement extent L1 Degree of Urbanisation attribute.

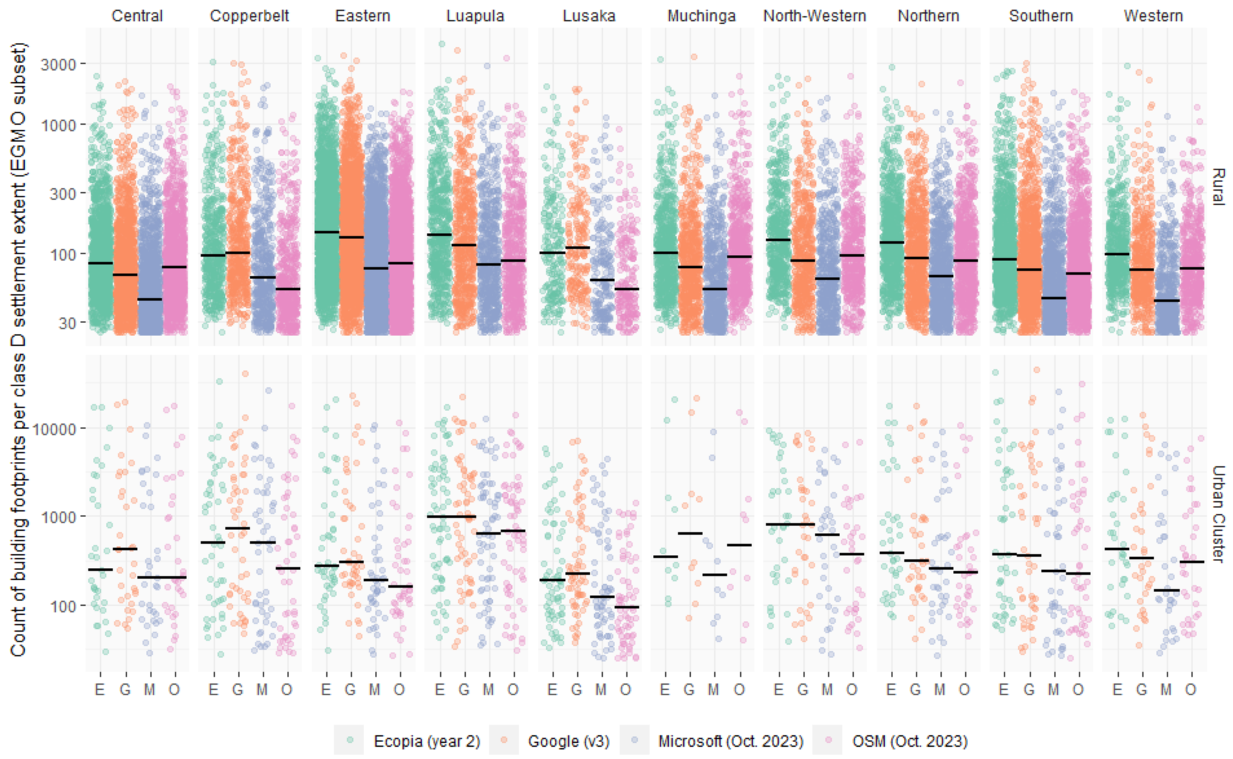

Figure A.4: For the subset of class D settlement extents that are found with all four building footprint datasets (EGMO), the count of potentially residential building footprints per settlement extent is shown with stratification per province and with rural/urban stratification. For each stratum, the median count of building footprints per settlement extent is shown by the horizontal black line. Rural/urban stratification is based on the settlement extent L1 Degree of Urbanisation attribute; given the small number of settlement extents classified as Urban Centres, this stratum has been omitted. **Note that the Y-axis for the Rural and Urban strata are different, both of which are on a log10 scale.**

Province	The count of class D settlement extents with each building footprint dataset, and the total count of potentially residential building footprints within those class D settlement extents							
	Ecopia (year 2)		Google (v3)		Microsoft (Oct. 2023)		OSM (Oct. 2023)	
	n sett. exts	n. BF	n sett. exts	n. BF	n sett. exts	n. BF	n sett. exts	n. BF
**Rural**								
Central	4,233	302,610	3,185	238,636	1,684	123,690	3,769	265,944
Copperbelt	1,338	132,328	1,562	158,682	818	82,963	620	56,546
Eastern	5,612	753,697	5,201	694,542	3,057	330,935	3,894	427,138
Luapula	1,314	205,256	1,093	169,372	772	101,833	834	117,310
Lusaka	1,021	98,959	1,129	105,006	558	49,450	295	22,897
Muchinga	1,808	172,273	1,469	136,935	689	60,405	1,817	164,842
North-Western	1,619	197,991	1,346	147,907	877	87,700	1,017	109,510
Northern	3,053	338,873	2,142	221,720	1,373	129,879	1,632	159,625
Southern	5,680	422,561	4,063	316,769	1,682	128,149	3,769	268,822
Western	4,404	275,342	3,050	188,634	921	59,648	2,178	127,751
TOTAL:	*30,082*	*2,899,890*	*24,240*	*2,378,203*	*12,431*	*1,154,652*	*19,825*	*1,720,385*
**Urban Cluster**								
Central	46	73,373	50	77,427	39	46,456	41	64,950
Copperbelt	72	111,317	94	137,929	68	93,173	51	68,687
Eastern	64	92,913	66	102,749	53	51,104	49	50,878
Luapula	75	177,355	80	181,228	67	109,563	71	121,369
Lusaka	128	50,849	153	65,278	107	40,280	70	16,723
Muchinga	20	48,471	26	52,334	10	15,520	25	40,530
North-Western	54	90,604	56	84,056	42	45,043	40	32,614
Northern	60	92,236	65	95,542	51	49,340	39	46,047
Southern	68	163,324	62	157,158	54	101,376	56	95,413
Western	51	73,561	51	65,537	35	35,991	43	34,860
TOTAL:	*638*	*974,003*	*703*	*1,019,238*	*526*	*587,846*	*485*	*572,071*
**Urban Centre**								
Central	2	111,625	2	119,973	2	86,282	2	96,676
Copperbelt	13	493,824	14	586,845	10	383,577	7	293,376
Eastern	1	47,551	1	54,940	1	31,000	1	23,974
Luapula	1	44,060	1	46,481	1	25,507	1	19,122
Lusaka	6	624,775	9	796,554	6	514,671	4	437,756
Muchinga	2	22,671	2	27,681	1	13,401	1	22,118
North-Western	1	92,739	1	106,216	1	64,280	1	6,123
Northern	1	50,531	1	54,826	1	29,431	1	21,632
Southern	1	31,068	1	33,182	1	18,574	1	21,468
Western	1	45,280	2	47,812	1	22,241	1	32,349
TOTAL:	*29*	*1,564,124*	*34*	*1,874,510*	*25*	*1,188,684*	*20*	*974,594*

Table A.5: For each building footprint dataset, the count of class D settlement extents and the total count of potentially residential building footprints within those class D settlement extents. Counts are stratified by province, with rural/urban stratification. Rural/urban stratification is based on the GRID3 v2.0 settlement extent L1 Degree of Urbanisation attribute.
